# Investigation of the Micromechanical Behavior of a Ti_68_Nb_7_Ta_3_Zr_4_Mo_18_ (at.%) High-Entropy Alloy

**DOI:** 10.3390/ma16145126

**Published:** 2023-07-20

**Authors:** Jin Wang, Qianli Ma, Hepeng Cheng, Hechun Yu, Suxiang Zhang, Huichao Shang, Guoqing Zhang, Wenbo Wang

**Affiliations:** 1 School of Mechatronics Engineering, Zhongyuan University of Technology, Zhengzhou 450007, China; 2021104101@zut.edu.cn (Q.M.); 2021104099@zut.edu.cn (H.C.); yuhechun1106@126.com (H.Y.); 3968@zut.edu.cn (S.Z.); sh_hc@126.com (H.S.); zgq@zut.edu.cn (G.Z.); wwb@zut.edu.cn (W.W.); 2 School of Mechanical and Transportation Engineering, Hunan University, Changsha 410082, China

**Keywords:** high-entropy alloy, molecular dynamics, dislocation evolution, strain rate, grain size

## Abstract

Intense research efforts are focused on the development of advanced high-entropy alloys intended for premium aerospace components and other applications, where high strength and good formability are crucial. The mechanical properties of these alloys are closely related to the phase transformation, dislocation evolution, and grain size, and these factors are affected by the deformation temperature. The response of the retained austenite to strain-induced martensitic transformation at various temperatures was studied in an advanced Ti_68_Nb_7_Ta_3_Zr_4_Mo_18_ (at.%) high-entropy alloy via molecular dynamics simulation. It was found that the Ti_68_Nb_7_Ta_3_Zr_4_Mo_18_ alloy changes from a single crystal to a polycrystal during the tensile process, and the transition of the Ti_68_Nb_7_Ta_3_Zr_4_Mo_18_ (at.%) high-entropy alloy from the BCC phase to the FCC phase occurs. At high temperatures and low strain rates, grain boundary slip is the main deformation mechanism, and at low temperatures and high strain rates, dislocation slip replaces grain boundary slip as the dominant deformation mechanism, which improves the strength of the alloy. Moreover, when the grain size is too small, the strength of the alloy decreases, which does not satisfy the fine grain strengthening theory and shows an inverse Hall–Petch relationship. This study offers a new compositional window for the additive manufactured lightweight high-strength material categories for various applications including the aerospace industry.

## 1. Introduction

High-entropy alloys (HEAs) were first proposed and named in 2004 [1]. It is generally believed that high-entropy alloys have four major effects: a high-entropy effect in thermodynamics, a hysteresis diffusion effect in dynamics, a lattice distortion effect in structure, and a “cocktail” effect in performance. Multiple principal elements have led to higher mixing entropy, which can inhibit segregation and even the combination of elements, thus promoting the formation of solid solutions of single FCC, BCC, and HCP phases [2,3,4]. Due to the diffusion-activated energy and interatomic binding force of different principal element atoms, these factors hinder the diffusion of atoms, making high-entropy alloys stable in a wide temperature range. In addition, the difference in atomic size will inevitably lead to more or less lattice distortion of the alloy, thus affecting its mechanical properties. Finally, the characteristics of different principal elements will interact with each other and influence each other to develop a synergistic performance effect. Compared to traditional metals, HEAs have excellent mechanical, chemical, and thermal properties, which are intended for premium aerospace components and other applications [5,6,7,8,9,10].

The excellent properties of HEAs have aroused the research interest of many scholars, from theory to experiments and molecular dynamics simulation. Taking into account the effect of strain rate, Li et al. conducted tensile simulations on an AlCrFeCuNi alloy and a CoNiFeAlCu alloy to study the mechanical properties of the alloys and the main mechanism of plastic deformation. The results showed that the AlCrFeCuNi alloy had not only high strength but also good plasticity. With the increase in strain, the AlCrFeCuNi alloy produced dislocation and stratification. Dislocation slip twins were the main deformation mechanism of the AlCrFeCuNi high-entropy alloy. Due to severe lattice distortion and high local strain, the CoNiFeAlCu alloy induced a phase transition from FCC to BCC. This phase transition mechanism can make the CoNiFeAlCu alloy obtain high ductility and high strength at the same time. In addition, this phase transition is mainly affected by the strain rate [11,12]. Geantă prepared an AlCrxFeCoNi (x = 0.2~2.0 at.%) high-entropy alloy with different Cr content and studied its microstructure and mechanical properties. The results showed that the content of Cr had an influence on the microstructure, grain size, and microhardness of the high-entropy alloy. With the change in Cr content, the microhardness changed from 389.6 to 562.6 HV_0.1_ [13]. Temperature also affects the properties of high-entropy alloys. Nutor et al. designed a face-centered cubic/B2 dual-phase HEA based on CoNiV by adjusting the composition and reported that the HEA maintained excellent strength–ductility synergism in a wide temperature range due to a variety of deformation mechanisms [14]. The stress and super-elasticity of high-entropy alloys with shape memory functions, such as NiTiHfPd, FeMnAlNi, etc., can also be affected by special temperature values [15,16]. In recent years, the properties of BCC phase high-entropy alloys with principal elements concentrated in refractory elements such as Ti, Zr, Ta, and Nb have been reported. These elements have the characteristics of thermal stability and high-temperature strength, and the density of the elements is low. Cr and Mo can improve the hardness and strength of the alloy, Ti can improve the plasticity and oxidation resistance of the alloy, and Zr can give consideration to both plasticity and strength; in addition, Zr can lead to the transformation of the phase structure of the alloy to diversification [17,18]. For example, the TaNbTaZrMo HEA has excellent yield strength and good biocompatibility, and the PBS medium showed excellent corrosion resistance comparable to Ti_6_Al_4_V, with a hardness and elastic modulus of 4.9GPa and 153GPa, respectively. The fracture mode and morphology of the alloy at room temperature show typical brittleness and finite plasticity [19]. In addition, the Mo element has a solid solution hardening effect, and its microhardness linearly scales with the Mo concentration, with an increase of ∼53% from 3.2 GPa to 4.9 GPa in the range of 0∼20 at.% Mo and an increment of 84 MPa per unit at.% Mo. This indicates that the Mo atoms generate a significant solid-solution hardening (SSH) effect on the quaternary TiZrNbTa alloy. The hardness and elastic modulus of the TiZrNbTaMox high-entropy alloy increase linearly with the concentration of the Mo element (0 ≤ x ≤ 20), while the ductility of the alloy decreases. When the concentration of the Mo element is 20%, the alloy has good corrosion resistance [20,21]. However, the influence of temperature and strain rate on the properties of the TiNbTaZrMo high-entropy alloy is still limited.

Thus, this research focuses on an HEA with Ti, Nb, Ta, Zr, and Mo, among which Ti, Nb, Ta, and Zr all have the function of improving the plasticity and ductility of the HEA. In addition, Ti and Zr are isomorphic in the solid state at room temperature, and Nb and Ta are also isomorphic; they have infinite compatibility and can be well fused. Mo can improve the hardness of HEAs. In order to maintain the hardness of the HEA while improving the plasticity, a Ti_68_Nb_7_Ta_3_Zr_4_Mo_18_ (at.%) HEA was chosen and simulated to obtain the effects of temperature, strain rate, and grain size on the mechanical properties of the Ti_68_Nb_7_Ta_3_Zr_4_Mo_18_ HEA. Moreover, the transformation process and deformation mechanism of the Ti_68_Nb_7_Ta_3_Zr_4_Mo_18_ (at.%) high-entropy alloy were also explored. This study will provide theoretical guidance for improving the service performance and structural regulation of HEAs.

## 2. Potential Functions and Simulation Methods

**Figure 1:**
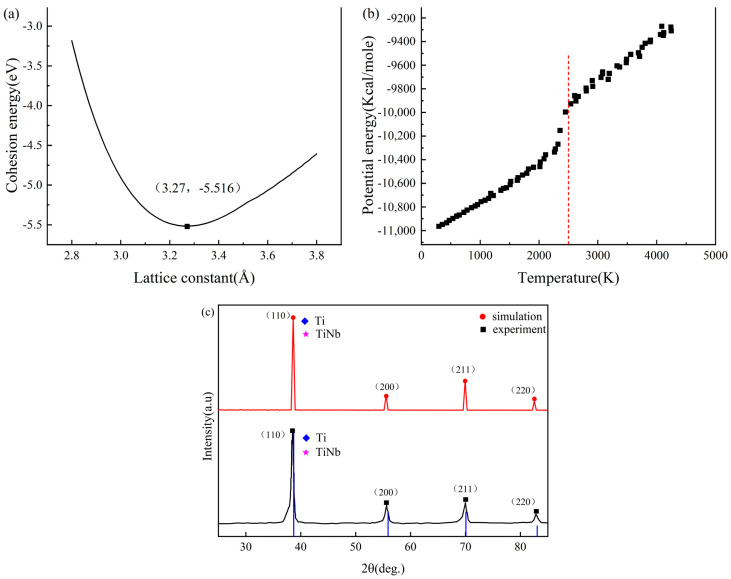
(**a**) Cohesion energy–lattice constant curve; (**b**) potential energy temperature curve; and (**c**) X-ray diffraction pattern.

Molecular dynamics simulations use potential functions to describe the interactions between atoms, and the accuracy of the potential functions determines the correctness of the results. At present, among the potential functions of metal elements, the embedded atom method (EAM) has been studied earlier and used more maturely. In this paper, the fitting potential function program developed by Zhou et al. was used to fit the required EAM potential function, and LAMMPS open-source code was used for all simulations [22]. Considering whether the fitting potential function is suitable for the alloy system, the potential function is verified by LAMMPS simulation of the average lattice constant, the melting point of the alloy, and X-ray diffraction (XRD) compared with the experiment. As shown in Figure 1a, the corresponding energy is the lowest when the lattice constant is 3.27 Å. In Figure 1b, the temperature corresponding to the sudden change in potential energy is the melting point, namely, 2500 K. Figure 1c shows the XRD experiment, simulation, and comparison of the diffraction pattern of the IDCC file numbered PDF-65-9438. The diffraction angles of each diffraction peak and the peak surface index are consistent. According to the systematic extinction law of X-ray diffraction, the BCC structure material will generate diffraction when the peak surface indices h, k, and l are added to an even number. The peak surface index of each diffraction peak of the high-entropy alloy is even, so the main structure of the high-entropy alloy is BCC. In addition, the first diffraction peak matches PDF-65-9438 well, and its phase is a TiNb compound. The above three groups of data show little difference from the measured values of Wang et al. [19].

**Figure 2:**
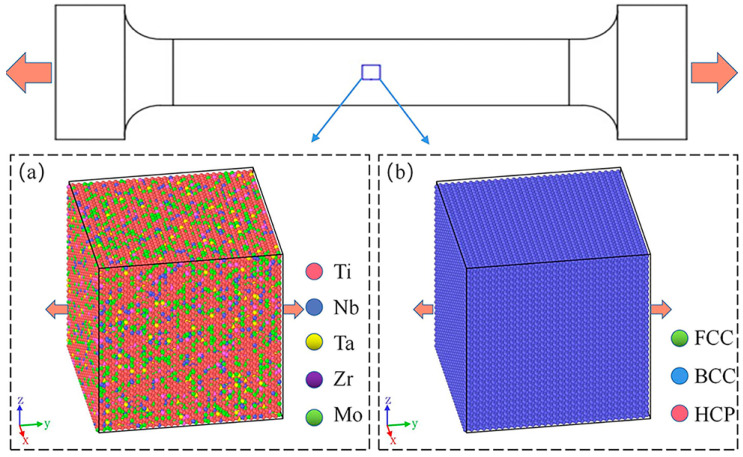
The Ti_68_Nb_7_Ta_3_Zr_4_Mo_18_ (at.%) HEA model: (**a**) atomic type and (**b**) CNA.

LAMMPS was used to construct the BCC structural model as shown in Figure 2. The size of the model was 30a × 30a × 30a, and five kinds of atoms (Ti, Nb, Ta, Zr, and Mo) were randomly assigned in proportion to the model, which contained a total of 54,000 atoms. To avoid the scale effect, the X, Y, and Z directions of the model were set as periodic boundary conditions (P P P). Conjugate gradient methods (CG) were used to minimize the energy of the model, and the model relaxed 50fs under isothermal and isobaric NPT. Finally, the model was stretched by 0.35 strains along the Y direction at different temperatures (77, 300, 700, and 1000 K) and strain rates (1 × 10^9^ s^−1^, 5 × 10^9^ s^−1^, 1 × 10^10^ s^−1^, 3 × 10^10^ s^−1^, and 5 × 10^10^ s^−1^). The whole process is completed in an isothermal isovolumic ensemble NVT. Ovito (3.8.5) software can visualize the simulation results. Common neighbor analysis (CNA) can distinguish the atomic structure, and the dislocation extraction algorithm (DXA) can track the dislocation motion [23].

## 3. Result

### 3.1. Mechanical Properties

**Figure 3:**
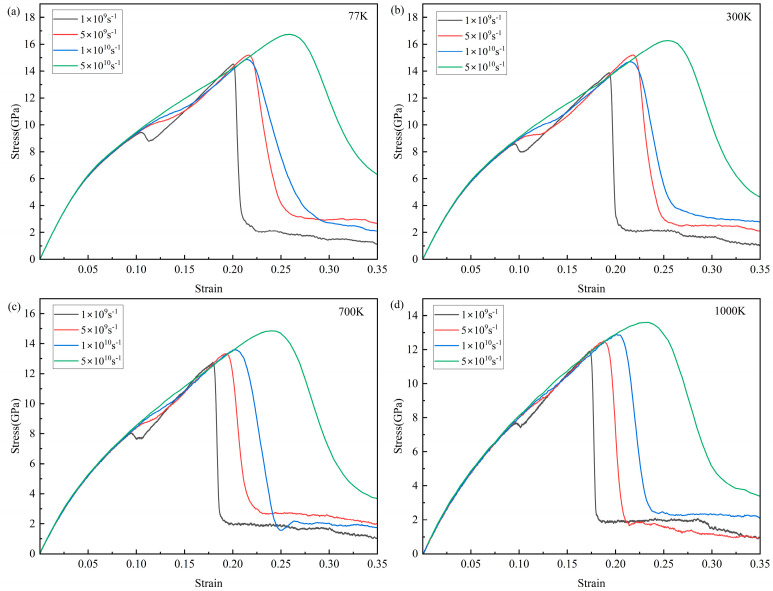
Stress–strain curves of the Ti_68_Nb_7_Ta_3_Zr_4_Mo_18_ (at.%) HEA at different temperatures (77, 300, 700, and 1000 K) and strain rates (1 × 10^9^ s^−1^, 5 × 10^9^ s^−1^, 1 × 10^10^ s^−1^, 3 × 10^10^ s^−1^, and 5 × 10^10^ s^−1^). (**a**) Stress-strain curves of 77 K at different strain rates; (**b**) Stress-strain curves of 300 K at different strain rates; (**c**) Stress–strain curves of 700 K at different strain rates; (**d**) Stress–strain curves of 1000 K at different strain rates.

**Figure 4:**
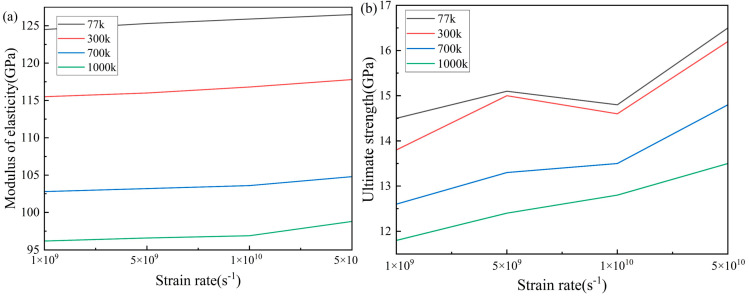
(**a**) Curves of elastic modulus with the strain rate at different temperatures and (**b**) curves of ultimate strength with the strain rate at different temperatures.

The stress–strain curves of the Ti_68_Nb_7_Ta_3_Zr_4_Mo_18_ (at.%) HEA stretched at different temperatures and strain rates are shown in Figure 3. The elastic modulus E of the alloy with high entropy can be obtained by fitting the slope of the 0–0.05 strain segment in the stress–strain curve. It can be seen from Figure 3 that the strain segments of curves from 0 to 0.05 basically coincide at the same temperature, so increasing the strain rate has no obvious effect on the elastic modulus of the HEA. The effect of the strain rate on the ultimate strength of the HEA is obvious, and the ultimate strength increases with the increase in the strain rate. The stress–strain curve has the same trend as the strain rate at different temperatures. When the strain rate was 1 × 10^9^ s^−1^ and the strain was 0.1, the curves all showed a downward change. With the increase in the strain, the curves continued to rise. After reaching the ultimate strength, the curves dropped rapidly. When the strain rate is increased, the curve does not decrease at 0.1 strain, but the rising rate of the curve after 0.1 strain first decreases and then increases. When the strain rate increases to 5 × 10^10^ s^−1^, the curve keeps rising steadily. In addition, the higher the strain rate, the slower the curve declines after reaching the ultimate strength. Figure 4 shows the changes in the elastic modulus and ultimate strength with the strain rate at different temperatures. It can be seen that temperature has a great influence on the properties of the Ti_68_Nb_7_Ta_3_Zr_4_Mo_18_ (at.%) HEA. With the increase in temperature, the elastic modulus and ultimate strength of the high-entropy alloy decrease obviously due to the softening effect of the high temperature. As shown in Figure 4a, with the increase in the strain rate, each curve had an upward trend, but the rise was slow, and the change in the elastic modulus was not obvious with the increase in the strain rate. As shown in Figure 4b, with the increase in the strain rate, the change in each curve was obvious, and the ultimate strength increased significantly. The ultimate strength decreases when the strain rate increases from 5 × 10^9^ s^−1^ to 1 × 10^10^ s^−1^ at 77 K and 300 K, but it is still larger than that at 1 × 10^9^ s^−1^. On the whole, there is a positive correlation between the ultimate strength and the strain rate. The elastic modulus E of the high-entropy alloy is 116.7 GPa at 300 K and the 1 × 10^10^ s^−1^ strain rate and the elastic modulus of the TiNbTaZrMo HEA is 153 GPa in the experiment. This is mainly caused by the different content of the Ti element. In this paper, the atomic concentration of Ti in the high-entropy alloy is about 68% [24].

### 3.2. Deformation Behavior

**Figure 5:**
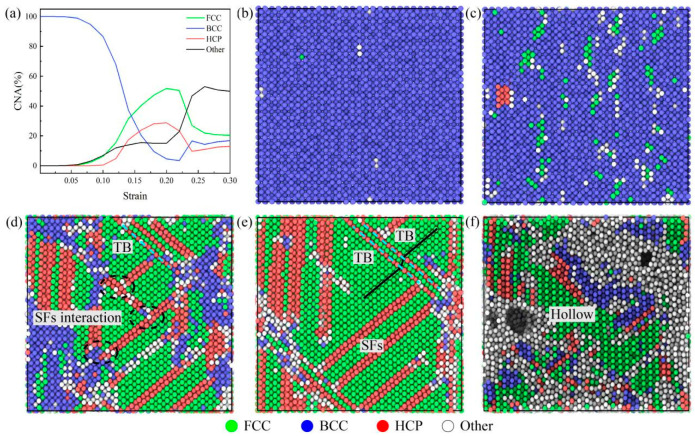
Structural snapshot of the Ti_68_Nb_7_Ta_3_Zr_4_Mo_18_ (at.%) HEA at different strains during the tensile process ((**a**): The proportion of individual structures in the tensile process; (**b**): ε = 0.05; (**c**): ε = 0.10; (**d**): ε = 0.15; (**e**): ε = 0.20; (**f**): ε = 0.25).

**Figure 6:**
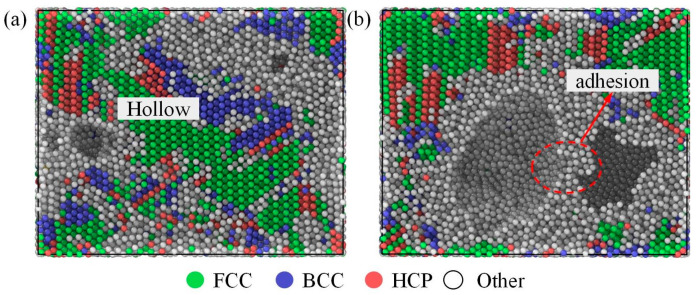
Snapshot of the Ti_68_Nb_7_Ta_3_Zr_4_Mo_18_ (at.%) HEA after stretching ((**a**) (100) and (**b**) (001) crystal face).

The different atomic structures in Ti_68_Nb_7_Ta_3_Zr_4_Mo_18_ (at.%) HEA crystals can be classified and colored by common neighbor analysis visualization. Figure 5 is a snapshot of the structural changes of the high-entropy alloy during the tensile process at the 1 × 10^10^ s^−1^ strain rate at a temperature of 300 K. Green, blue, and red represent the FCC, BCC, and HCP structures, respectively, and white represents atoms that do not satisfy any of the basic structures. It is well known that a single layer composed of HCP atoms means a twin boundary (TB), and a double layer of HCP means stacking faults (SFs). The structure of high-entropy alloy changes with the increase in the tensile load. As shown in Figure 5b–e, the initial BCC structure of the high-entropy alloy is transformed into an FCC structure, accompanied by a crisscross HCP structure and disordered white atoms. With the increase in strain, the HCP structures gradually increase and fuse with BCC phase boundaries, as shown in the black dotted line in Figure 5d. When stretched to 0.2 strain, almost all of the BCC phase was transformed into the FCC phase, and twins appeared in the black line art of Figure 5e. When the tensile load continues to be applied, the high-entropy alloy generates a cavity at the grain boundary and fractures, as shown in Figure 5f. Therefore, the grain of the high-entropy alloy also changes during the tensile process. In Figure 5a, it can be seen more directly that the FCC structure increases rapidly at the expense of the BCC structure. At 0.2 strain, the FCC structure reaches its maximum content, accounting for about 51%. As the tension continues, the hollow formed by the high-entropy alloy gradually increases. Figure 6 is a snapshot of the failure of the high-entropy alloy, and there is a certain adhesion on the (001) crystal plane. It can be seen that this type of fracture is more like the dimple produced when the plastic fracture occurs, so this high-entropy alloy has a certain degree of toughness.

**Figure 7:**
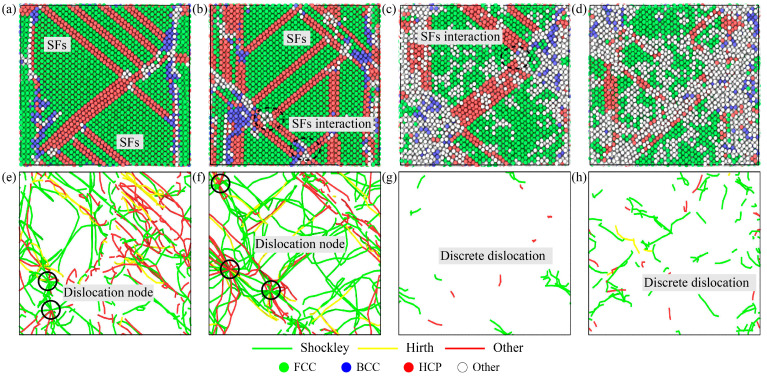
Structural distribution and dislocation distribution under different tensile conditions (**a**,**e**) 77 K and 1 × 10^9^ s^−1^, (**b**,**f**) 77 K and 1 × 10^10^ s^−1^, (**c**,**g**) 1000 K and 1 × 10^9^ s^−1^, and (**d**,**h**) 1000 K and 1 × 10^10^ s^−1^.

In order to reveal the influence of temperature and strain rate on the deformation mechanism of the high-entropy alloy, the structural changes and dislocation evolution of the high-entropy alloy under different tensile conditions were studied. After stretching to ultimate strength, the structure and dislocation of the high-entropy alloy at 77 K and 1000 K temperatures are shown in Figure 7a–d,e–h, respectively. It can be seen that the deformation of the high-entropy alloy is mainly divided into grain boundary slip and dislocation slip. When the temperature rises, the atoms inside the alloy move violently, and some atoms at the grain boundaries break away from their original positions and become disordered, resulting in grain boundary damage, as shown in the black dotted line in Figure 7c,d. In addition, there are some differences in dislocation density at different temperatures and strain rates. At 77 K, the dislocation densities of 1 × 10^9^ s^−1^ and 1 × 10^10^ s^−1^ are 4.6 × 10^17^ m^−2^ and 5 × 10^17^ m^−2^, respectively. At 1000 K temperature, the dislocation densities of 1 × 10^9^ s^−1^ and 1 × 10^10^ s^−1^ are 2.5 × 10^16^ m^−2^ and 1 × 10^17^ m^−2^, respectively. The green line represents 1/6[1 1 2] Shockley, yellow represents 1/3[1 0 0] Hirth, and red represents the other. It is clear that the main type of dislocation in the alloy is Shockley dislocation, which is a continuous long dislocation at low temperatures and a discrete short dislocation at high temperatures. Dislocations occur mainly within the grain, and most remain parallel to each other. The grain boundary has a certain hindrance effect on the dislocation slip, so at the grain boundary, the dislocations accumulate and wrap to form dislocation nodes, as shown in the black coils in Figure 7e,f. With the increase in the strain rate, the length of the dislocation line increases, more dislocation entanglement occurs at the grain boundary, and the dislocation density increases. At high temperatures, the phenomenon of dislocation entanglement is not obvious, but the length and density of the dislocation line increase significantly, as shown in Figure 7g,h.

### 3.3. The Effect of Grain Size on the Mechanical Properties

**Figure 8:**
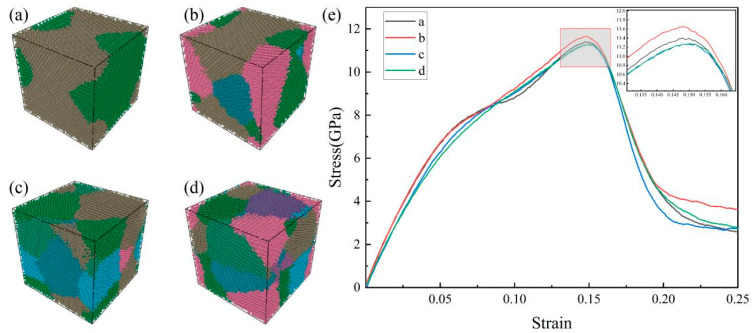
HEA model with a different number of grains. Grain number: (**a**) 2, (**b**) 4, (**c**) 6, and (**d**) 8. (**e**) Stress–strain curves.

When the strain rate of 300 K is 1 × 10^10^ s^−1^, the monocrystalline is transformed into polycrystalline in addition to molecular structure transformation. In order to study the effect of grain size on the mechanical properties, the Voronoi structure method was used to modify the model [25,26], as shown in Figure 8a–d. All the models were 10 × 10 × 10 nm^3^ in size and contained different quantities of randomly oriented particles, which were controlled by this method. Figure 8e shows the stress–strain curve of the HEA after the tensile process. It can be seen that in the strain 0–0.05 stage, the rate of curve rise is different, so the elastic modulus is affected by the grain size. When the number of grains increases, the grain size decreases, and the elastic modulus also decreases. When the strain reaches about 0.15, the alloy with high entropy reaches the ultimate strength. The ultimate strength of the alloy with the number of grains at 4 is the highest, the ultimate strength of the alloy with the number of grains at 2 is only second, and the ultimate strength of the alloy with the number of grains at 6 and 8 is the lowest and basically the same. Generally, fine grain strengthening can be expressed by the Hall–Petch relationship, that is, the strength of the material is inversely proportional to the grain size. It is obvious that the appeal result does not fully satisfy this relationship. When the grain size is reduced to a certain extent, the inverse Hall–Petch phenomenon appears [27].

**Figure 9:**
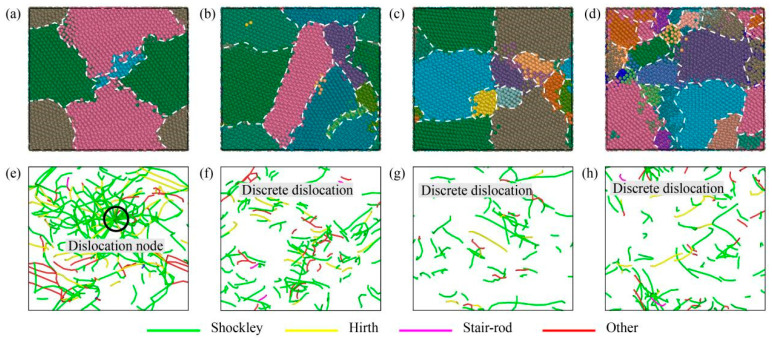
HEA structure and dislocation with different grains at 0.15 tensile strain: (**a**–**d**) HEA structure and (**e**–**h**) HEA dislocation.

In order to further investigate the cause of the inverse Hall–Petch phenomenon, a snapshot of the grain and dislocation with the tensile strain of the high-entropy alloy at 0.15 was taken, as shown in Figure 9. After stretching, the number of grains increases further, and the grain size becomes smaller. For dislocation, in high-entropy alloys with relatively large grain sizes, dislocation lines are denser and longer, and entanglement to form dislocation knots is more likely, as shown in Figure 9e. However, in the high-entropy alloy with a relatively small grain size, the length of the dislocation line is shorter and there is not much winding, forming a discrete state distribution, as shown in Figure 9e–h. In addition, a small number of 1/6[1 1 0] stair-rod dislocations occur in polycrystalline stretching compared to single-crystal stretching.

## 4. Discussion

**Figure 10:**
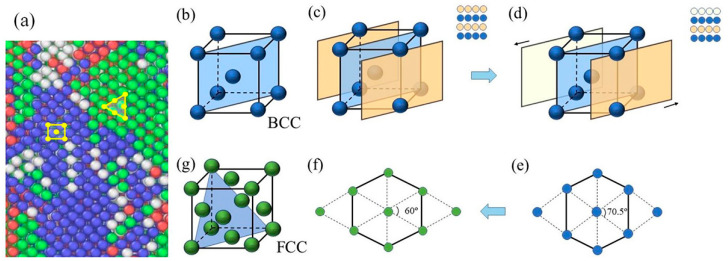
Schematic diagram of phase transition from BCC to FCC. (**a**) Snapshot of the transition from BCC to FCC (**b**) Structure of BCC; (**c**–**f**) The transition from BCC to FCC; (**g**) Structure of FCC.

The Ti_68_Nb_7_Ta_3_Zr_4_Mo_18_ (at.%) HEA model underwent BCC–FCC phase transition in uniaxial *Y*-axis stretching along the NVT system, which is somewhat different from the single crystal Fe model studied by Ma et al. [28], which underwent BCC–FCC phase transition under uniaxial compression along the *X*-axis. During the stretching process, the transition from BCC to FCC is divided into two stages. First, under tensile load, the BCC close-packed surface atoms change from “ABABAB” stacking mode to “ABCABCABC” stacking mode, as shown in Figure 10c,d, and the lattice angle changes from 90° to 70.5°. Then, the atoms in the BCC close-packed surface rearrange, and the lattice Angle becomes 60°, as shown in Figure 10e,f. Therefore, the BCC structure is transformed into an FCC structure, as shown in Figure 10g. Figure 10a shows the microscopic magnification of the BCC and FCC phases at 0.15 strain. The whole phase transition process is similar to the transition from BCC to FCC studied by Ding et al. and satisfies the KS relationship [29,30].

As can be seen from the results in Summary I confirm 3.2 of this paper, obvious grain boundary slip occurs in the high-entropy alloy at high temperatures. Due to the high temperature, the thermal motion of atoms is intensified, and a large number of disordered atoms are formed from the original position of atoms, and these disordered atoms are mostly concentrated near the crystallization, as shown in Figure 7c,d. At low temperatures, this phenomenon is not obvious, and no large number of disordered atoms are produced, as shown in Figure 7a,b. However, at low temperatures, a large number of dislocations are generated, which slip within the grain and gather and intertwine at the grain boundaries to form dislocation knots, as shown in Figure 7e,f. The strain rate has a great influence on dislocation. By comparing Figure 7e,f (or Figure 7g,h), it can be found that more dislocations and entanglement occur at higher strain rates. Therefore, at high temperatures, grain boundary slip is more serious and plays a leading role in deformation. At low temperatures and high strain rates, dislocation slip is more serious and dominates the deformation mechanism. This is consistent with the conclusion of Li et al. [31], who studied the influence of temperature and strain rate on the deformation mechanism of high-entropy alloys.

It is generally believed that dislocation occurs in the deformation process of materials, and dislocations intertwine with each other to form dislocation knots, resulting in increased dislocation density and relatively difficult dislocation movement. Because the free travel of dislocation movement decreases, the interaction between dislocation hinders the slip movement of dislocation, and the stronger the material resistance to deformation, the higher the strength of the material. However, when the grain size is less than a certain degree, it is difficult for the dislocation to move within the grain, and the phenomenon of intertwining will not appear. In addition, grain boundaries are more likely to break down during deformation due to the small grain size. The above two reasons will lead to the reduction of material strength and reverse the Hall–Petch phenomenon at the realization point.

## 5. Conclusions

The micromechanical properties of a Ti_68_Nb_7_Ta_3_Zr_4_Mo_18_ (at.%) HEA were studied by the molecular dynamics method, and the effects of temperature, strain rate, and grain size on the mechanical properties were considered. The following conclusions were obtained.

1. High temperatures can soften the alloy and reduce the elastic modulus and ultimate strength of the alloy. The strain rate has little effect on the elastic modulus of the high-entropy alloy but a great effect on the ultimate strength of the high-entropy alloy, that is, the higher the strain rate, the greater the ultimate strength.
2. The transition from an FCC structure to a BCC structure occurs during the tensile process of the Ti_68_Nb_7_Ta_3_Zr_4_Mo_18_ (at.%) HEA. The main deformation mechanism at high temperatures is grain boundary slip, while the main deformation mechanism at low temperatures and high strain rates is dislocation slip.
3. The grain size of the Ti_68_Nb_7_Ta_3_Zr_4_Mo_18_ (at.%) HEA is too small due to the internal dislocation of grain growth and slippage, and with the instability of grain boundaries, the strength of the alloy will reverse the Hall–Petch law phenomenon.

## Data Availability

All data used to support the findings of this study are included within the article.
